# *Citrobacter rodentium* is an Unstable Pathogen Showing Evidence of Significant Genomic Flux

**DOI:** 10.1371/journal.ppat.1002018

**Published:** 2011-04-07

**Authors:** Nicola K. Petty, Theresa Feltwell, Derek Pickard, Simon Clare, Ana L. Toribio, Maria Fookes, Kevin Roberts, Rita Monson, Satheesh Nair, Robert A. Kingsley, Richard Bulgin, Siouxsie Wiles, David Goulding, Thomas Keane, Craig Corton, Nicola Lennard, David Harris, David Willey, Richard Rance, Lu Yu, Jyoti S. Choudhary, Carol Churcher, Michael A. Quail, Julian Parkhill, Gad Frankel, Gordon Dougan, George P. C. Salmond, Nicholas R. Thomson

**Affiliations:** 1 Wellcome Trust Sanger Institute, Wellcome Trust Genome Campus, Hinxton, Cambridge, United Kingdom; 2 Department of Biochemistry, University of Cambridge, Cambridge, United Kingdom; 3 Centre for Molecular Microbiology and Infection, Division of Cell and Molecular Biology, Imperial College London, London, United Kingdom; Yale University, United States of America

## Abstract

*Citrobacter rodentium* is a natural mouse pathogen that causes attaching and effacing (A/E) lesions. It shares a common virulence strategy with the clinically significant human A/E pathogens enteropathogenic *E. coli* (EPEC) and enterohaemorrhagic *E. coli* (EHEC) and is widely used to model this route of pathogenesis. We previously reported the complete genome sequence of *C. rodentium* ICC168, where we found that the genome displayed many characteristics of a newly evolved pathogen. In this study, through PFGE, sequencing of isolates showing variation, whole genome transcriptome analysis and examination of the mobile genetic elements, we found that, consistent with our previous hypothesis, the genome of *C. rodentium* is unstable as a result of repeat-mediated, large-scale genome recombination and because of active transposition of mobile genetic elements such as the prophages. We sequenced an additional *C. rodentium* strain, EX-33, to reveal that the reference strain ICC168 is representative of the species and that most of the inactivating mutations were common to both isolates and likely to have occurred early on in the evolution of this pathogen. We draw parallels with the evolution of other bacterial pathogens and conclude that *C. rodentium* is a recently evolved pathogen that may have emerged alongside the development of inbred mice as a model for human disease.

## Author Summary

The pathogenic bacterium *Citrobacter rodentium* naturally infects mice using a mechanism similar to those employed by certain strains of *E. coli* that cause severe gastro-intestinal infections in humans. As such it is an important model organism for human disease research. We previously sequenced the genome of *C. rodentium* strain ICC168 and found that it had many features in common with other bacterial pathogens that have recently adapted to live in a new environment and colonise new hosts. In this study, we sequenced the genome of an additional strain of *C. rodentium* that was independently isolated on a different continent, and found that the two strains were remarkably similar. In addition, we investigated several *C. rodentium* isolates and showed that the genome is unstable, existing in multiple conformations within a single population due to genomic inversions, recombination between repetitive sequences, and horizontally acquired DNA that is mobile within the genome. We conclude that *C. rodentium* recently evolved to become a pathogen of mice, possibly concurrently with the development of mouse models for human diseases, and that it is still adapting to its new environment.

## Introduction

The genomes of enteric bacteria have been shown to be dynamic entities through gene acquisition and loss. It is clear that these genomes consist of a highly conserved core inter-dispersed with a continually evolving accessory genome. Genome flux can have a profound effect on a particular organism, in many instances it is associated with adaptation to different niches and may eventually come to define different isolates, pathotypes or even species. Genome flux can occur by Horizontal Gene Transfer (HGT) through processes such as transformation, bacteriophage mediated transduction and conjugation. In addition to gene gain, gene loss through deletions, rearrangements and the accumulation of point mutations are also major inputs to genome flux and have been linked to host adaptation, for example in *Salmonella* Typhi and *Yersinia pestis* [1], [2] whereby functions important for the previous lifestyle are no longer preserved through selection and so accumulate random mutations.

The non-motile, Gram-negative enteric bacterium *Citrobacter rodentium* is a natural mouse pathogen. It is the causative agent of transmissible murine colonic hyperplasia, and is responsible for high mortality in suckling mice [3]–[5]. *C. rodentium* is a member of a family of bacterial pathogens that induce intestinal attaching and effacing (A/E) lesions, which are characterised by intimate bacterial adherence to host intestinal epithelial cells, effacement of microvilli, and reorganisation of the host actin cytoskeleton to form pedestal-like extensions of epithelial cells beneath the adherent bacteria [6]. Gastrointestinal colonisation and formation of A/E lesions are mediated by a pathogenicity island called the locus of enterocyte effacement (LEE), which is conserved among A/E bacteria [6], [7]. As the only known A/E pathogen to naturally infect mice, *C. rodentium* is a valuable model organism for studying colonisation, virulence factors and modes of pathogenesis of the clinically significant human A/E pathogens enteropathogenic *E. coli* (EPEC) and enterohaemorrhagic *E. coli* (EHEC) [6], [8], [9].

Different *C. rodentium* isolates from mouse and hamster colony disease outbreaks in Japan and the USA in the 1960s, 70s and 80s were originally classified as either atypical mouse-pathogenic *E. coli* (MPEC) [10]–[12] or atypical *Citrobacter freundii* (later reclassified as *Citrobacter* genomospecies 9) [13]–[16]. However, subsequent genetic and biochemical analyses of these independently isolated strains suggested they were of clonal origin and they were all reclassified as *Citrobacter rodentium* [17], [18].

We previously determined the whole genome sequence of *C. rodentium* strain ICC168, a derivative of a strain isolated from a disease outbreak in Swiss-Webster mice at Yale University School of Medicine, USA, in 1972, originally designated *Citrobacter freundii* biotype 4280 (ATCC 51459) [13], [19]. ICC168, together with strain DBS100 which originates from the same source [5], [9], are the most widely studied *C. rodentium* isolates. We showed that the genome of ICC168 displayed features associated with bacteria that have recently passed through an evolutionary bottleneck, including a large number of pseudogenes and IS elements [20]. Here, we present the detailed investigation of genomic flux in *C. rodentium* with a focus on the impact that mobile genetic elements have had on the genome evolution of *C. rodentium* and demonstrate that the genome of this pathogen is unstable. To show that this is a consistent feature of the species, we determined the genome sequence of an additional *C. rodentium* strain, EX-33 (originally classified as MPEC [11]), which was isolated from a spontaneous outbreak of disease in a CF-1 mouse colony at the Institute of Medical Science, University of Tokyo, Japan in 1981 (K. Itoh, personal communication) and showed differences in levels of colonisation and disease pathology compared to DBS100 [17]. We describe the effect the observed genome rearrangements have on the ability of *C. rodentium* to infect the murine host and relate these findings to the evolution of this important model pathogen.

## Results and Discussion

### *C. rodentium* ICC168 is representative of the species

To ensure that the genome of ICC168 was representative of the *C. rodentium* species we determined the whole genome draft sequence of the independently isolated strain EX-33 using 454 and Illumina sequence data to construct a combined *de novo* assembly (see methods). The genome of EX-33 was found to be remarkably similar to ICC168. Despite being merely a draft sequence, the genome of EX-33 differed from ICC168 by just 177 single nucleotide polymorphisms (SNPs), only 43 of which were high-quality validated SNPs, and two deletions (details of all the differences between ICC168 and EX-33 are listed in Tables 1 and S1).

**Table 1:** Gene differences between *C. rodentium* strains EX-33 and ICC168.

CDS ID*	Product	EX-33	ICC168	Comment
ROD_02831	IS*102* transposase	absent	present	IS*102* insertion in ICC168
ROD_02841	Hypothetical prophage protein (CRP28)	intact	pseudo	Disrupted by IS*102* insertion in ICC168
ROD_05221	IS*102* transposase	absent	present	IS*102* insertion in ICC168
ROD_05231	Putative transposase	intact	pseudo	Disrupted by IS*102* insertion in ICC168
ROD_15301	Hypothetical protein	pseudo	intact	N-terminal truncated by 932 bp deletion in EX-33
ROD_20681	IS*102* transposase	absent	present	IS*102* insertion in ICC168
ROD_28451	IS*102* transposase	absent	present	IS*102* insertion in ICC168
ROD_28951	IS*102* transposase	absent	present	IS*102* insertion in ICC168
ROD_28941	Putative exported protein	intact	pseudo	Disrupted by IS*102* insertion in ICC168
ROD_29281	IS*102* transposase	absent	present	IS*102* insertion in ICC168
ROD_29471	IS*102* transposase	absent	present	IS*102* insertion in ICC168
ROD_29461/81	Hypothetical protein	intact	pseudo	Disrupted by IS*102* insertion in ICC168
ROD_31511	IS*102* transposase	absent	present	IS*102* insertion in ICC168
ROD_31521	Hypothetical protein	intact	pseudo	Disrupted by IS*102* insertion in ICC168
ROD_33401	IS*102* transposase	absent	present	IS*102* insertion in ICC168
ROD_33391	Hypothetical protein	intact	pseudo	Disrupted by IS*102* insertion in ICC168
ROD_35171	IS*102* transposase	absent	present	IS*102* insertion in ICC168
ROD_42661	ATP-binding protein of dipeptide ABC transporter	pseudo	intact	Truncated by premature stop codon due to SNP in EX-33
ROD_45501	IS*102* transposase	absent	present	IS*102* insertion in ICC168
ROD_48241	Hypothetical protein	pseudo	intact	C-terminal truncated by 4392 bp deletion in EX-33
ROD_48251	Putative membrane protein	absent	present	4392 bp deletion in EX-33
ROD_48261	Putative LysR-family transcriptional regulator	absent	present	4392 bp deletion in EX-33
ROD_48271	Putative transport protein	pseudo	intact	N-terminal truncated by 4392 bp deletion in EX-33
ROD_50641	IS*102* transposase	absent	present	IS*102* insertion in ICC168
ROD_50631	Putative fimbrial usher protein	intact	pseudo	C-terminal truncated by IS*102* insertion in ICC168, intact in Ex33
ROD_50632	Putative fimbrial protein	present	absent	Deletion in ICC168 due to IS*102* insertion
ROD_50633	Putative fimbrial protein	present	absent	Deletion in ICC168 due to IS*102* insertion
ROD_50651	Putative fimbrial adhesin	intact	pseudo	N-terminal truncated by IS*102* insertion in ICC168
ROD_p2_471	IS*102* transposase	absent	present	IS*102* insertion in ICC168
ROD_p2_461	Putative conjugal transfer protein TriD	intact	pseudo	Disrupted by IS*102* insertion in ICC168
ROD_p4_51	Hypothetical protein	pseudo	intact	Truncated by frameshift mutation due to SNP in EX-33

*Names used are those in the ICC168 genome.

The high conservation also included all of the mobile genetic elements, including prophages, insertion sequence (IS) elements and genomic islands (GI), all of which are present and found at exactly the same sites in both genomes, the one exception being IS*102* which is absent from EX-33 but expanded to 13 copies in ICC168.

The insertions of IS*102* elements have disrupted nine single genes and a fimbrial operon in ICC168, each of which are found intact in EX-33 (Table 1). Conversely, there are two deletions in EX-33 compared to ICC168 (Table 1). The first is a 932 bp deletion, which has resulted in the truncation of ROD_15301 encoding a hypothetical protein. The second is a 4392 bp deletion in EX-33 that has deleted two genes (ROD_48251 and ROD_48261) and truncated two other genes (ROD_48241 and ROD_48271) of unknown function. In ICC168, the sequences corresponding to both of these EX-33 deletions are flanked by 2 bp and 6 bp direct repeats respectively. Although these repeats are short sequences, the data suggests that in both cases the deletions were due to site-specific recombination.

The data presented here is consistent with there being a clonal origin for this species and provides evidence of continued functional gene loss in both of these *C. rodentium* strains.

### Evidence of large-scale genomic rearrangements

**Figure 1:**
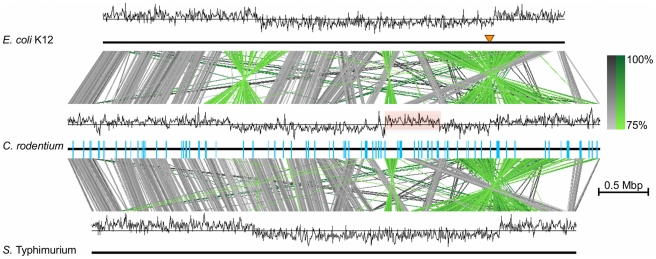
Comparison of the genome of *C. rodentium* with related bacteria. Genome comparison of the DNA sequences of *C. rodentium* ICC168 (middle) with *E. coli* K12 MG1655 (U00096, top) and *Salmonella* Typhimurium LT2 (AE006468, bottom). Grey shading between two genomes indicates regions of nucleotide similarity (BLASTN matches with a minimum length of 1000 bp) between sequences on the same strand, green shading highlights where the matching sequences are inverted with respect to each other (percentage identity is indicated). The locations of IS elements in the *C. rodentium* genome are shown as blue bars. An orange pointer indicates the origin of replication (*oriC*) in the *E. coli* genome. The GC Deviation (G−C)/(G+C) plot is shown above each genome (window size 1000 bp) with the switch in GC deviation in *C. rodentium* highlighted by red shading. The scale bar indicates genome length. This figure was produced using Easyfig [67].

The genomic architecture of *C. rodentium* contains a large intra-replichore inversion of approximately 0.5 Mb in the genome of ICC168, resulting in a switch in GC deviation (Figure 1). We used PCR to show that the same inversion is also present in EX-33. GC deviation switches are usually only seen at the origin and terminus of replication in bacteria [21] as can be seen in the GC deviation plots for the genomes of both *E. coli* K-12 and *Salmonella* Typhimurium LT2 [22], [23] (Figure 1). In addition, it was evident from whole genome comparisons that whilst *C. rodentium* ICC168 shares significant conservation in genome synteny with *E. coli* and *Salmonella*, there are many chromosomal inversions and rearrangements in the genome including two large inversions spanning the origin and terminus of replication, the latter being identical to an inversion found in *S.* Typhimurium LT2 (Figure 1). Inversions over the terminus are the most common form of large genomic rearrangement detected in enteric bacteria, and homologous recombination between rRNA operons resulting in such rearrangements have previously been observed in host-specific *Salmonella* species [24]. However, unlike in *Salmonella*, recombination between rRNAs did not explain the genome rearrangements found in *C. rodentium*, which were largely flanked by IS elements (Figure 1).

The IS elements found in ICC168 belong to a diverse range of IS families. However, only 8 types of IS element comprise 66% of the 113 insertions, indicating extensive IS expansion, particularly for IS*Cro1* (Table 2). Interestingly, IS elements or IS element-related inverted repeats also flank 6 of the 17 GIs identified in the ICC168 genome [20] and over half of the IS elements in the ICC168 genome are located on other mobile genetic elements rather than on the chromosomal backbone (Table 2). This highly biased distribution of IS elements is similar to that observed in EHEC O157 genomes [25]. These data, taken together with previous findings [20], [26], suggest that IS elements are associated with chromosomal rearrangements, and horizontal gene transfer facilitating the incorporation of novel gene functions into the *C. rodentium* genome.

**Table 2:** Classification and location of IS elements in *C. rodentium*.

	Total number	Genomic location			
IS element^a^	(of which are remnants)	Chromosomal backbone	Genomic Island	Prophage	Plasmid
IS*102* b	13	9	2	1	1
IS*200* family (IS*200E*-like)	1 (1)	1			
IS*200* family (IS*200F*-like)	2 (2)	1	1		
IS*200* family (IS*200I*-like)	3 (2)	2	1		
IS*21* family (IS*100kyp*-like)	1 (1)		1		
IS*21* family (IS*Sso4*-like)	1 (1)		1		
IS*256* family (IS*285*-like)	1 (1)		1		
IS*256* family (IS*1414*-like)	1 (1)				1
IS*3*	1		1		
IS*3* family (IS*3G*-like)	1 (1)		1		
IS*3* family (IS*911*-like)	3 (3)	1	2		
IS*3* family (IS*Eam1*-like)	1 (1)		1		
IS*3* family (IS*Ec11*-like)	1 (1)		1		
IS*3* family (IS*Ehe3*-like)	2 (2)		2		
IS*3* family (IS*Sen1*-like)	2 (2)		2		
IS*4*	1 (1)		1		
IS*4* family (IS*Sfl1*-like)	1 (1)		1		
IS*66* family (IS*Ec23*-like)	1 (1)		1		
IS*679*	1		1		
IS*91* family IS*91*-like	1 (1)				1
IS*Cro1*	24 (2)	14	7	2	1
IS*Cro2*	3	3			
IS*Cro3*	14 (3)	9	3	2	
IS*Cro4*	13	13			
IS*Cro5*	5	5			
IS*Cro6*	6 (4)	6			
IS*Ec14*	6	2	3	1	
IS*Ec23*	1 (1)		1		
Unclassified IS	2 (1)	1		1	
**Total = 29 IS elements**	**113 (34 remnants** c **)**	**67**	**35**	**7**	**4**

a In addition to IS elements, there are 12 unclassified transposases (five are located on the chromosome and seven on GIs), nine of which are remnants.

b Unique to ICC168, not in EX-33 genome.

c The number of remnants was previously reported incorrectly [20]. The correct number of IS element remnants is 34 (32 on the chromosome and 2 on plasmid pCROD1).

### Evidence for ongoing genome instability in *C. rodentium*

**Figure 2:**
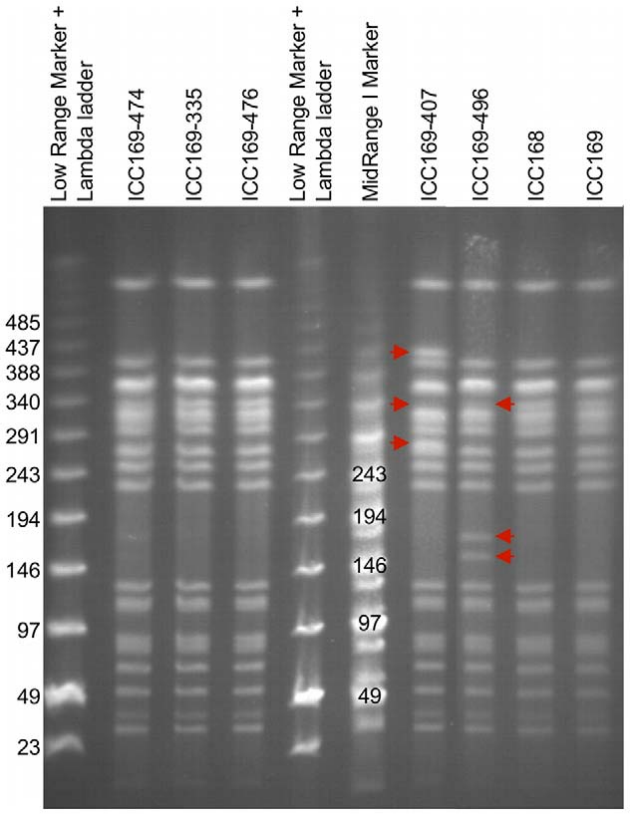
PFGE profile of different *C. rodentium* isolates. PFGE generated after *Xba*I cleavage of genomic DNA isolated from different strains and isolates of *C. rodentium*. Strain ICC168 shows the same PFGE pattern as for ICC169, ICC169-474, ICC169-335 and ICC169-476. Two isolates displayed significant differences in their PFGE profiles (indicated by red arrows); ICC169-407 has a band missing at approximately 340 kb and additional bands of approximately 280 kb and 420 kb; ICC169-496 is also missing the 340 kb band and has two extra bands between 145 and 200 kb. Markers are from New England BioLabs. Band sizes are indicated in kb.

To investigate if the observed genomic architecture of *C. rodentium* was stable, we analysed the PFGE profiles for *C. rodentium* strain ICC168 and derivatives of that strain representing the majority of *C. rodentium* isolates used in our laboratories (Table 3). This analysis revealed that ICC168 exhibited the same PFGE pattern as ICC169, ICC169-474, ICC169-335 and ICC169-476. However, isolates ICC169-407 and ICC169-496 displayed significant differences in their PFGE profiles, compared to ICC168 (Figure 2).

**Table 3:** Bacterial strains used in this study.

Strain	Description	Comment	Reference
***Citrobacter rodentium***			
EX-33	Previously *Escherichia coli* O115a,c:K(B) strain EX-33	pCROD1^+^	[11]
ICC168	Previously *Citrobacter freundii* biotype 4280 (ATCC 51459)	pCROD1^+^	[13]
ICC169	Spontaneous Nal^R^ derivative of ICC168, original stock	PFGE profile same as for ICC168, pCROD1^+^	[66]
ICC169-335	Dougan laboratory isolate, previously ICC169	PFGE profile same as for ICC168, pCROD1^−^	This study
ICC169-407	Dougan laboratory isolate, previously ICC169	Differences in PFGE profile compared to ICC168, pCROD1^+^	This study
ICC169-474	Frankel laboratory isolate, previously ICC169	PFGE profile same as for ICC168, pCROD1^+/−^	This study
ICC169-476	Salmond laboratory isolate, previously ICC169	PFGE profile same as for ICC168, pCROD1^−^	This study
ICC169-496	Dougan laboratory isolate, previously ICC169	Differences in PFGE profile compared to ICC168, pCROD1^−^	This study
ICC169c3	Isolated from ICC169 infected mouse ‘c’ faeces 3 days post infection	PFGE profile same as for ICC168	This study
ICC169c13	Isolated from ICC169 infected mouse ‘c’ faeces 13 days post infection	PFGE profile same as for ICC168	This study
ICC169c14cae	Isolated from ICC169 infected mouse ‘c’ caecum 14 days post infection	PFGE profile same as for ICC168	This study
ICC169c14col	Isolated from ICC169 infected mouse ‘c’ colon 14 days post infection	PFGE profile same as for ICC168	This study
ICC169a3	Isolated from ICC169 infected mouse ‘a’ faeces 3 days post infection	PFGE profile same as for ICC168	This study
ICC169a15	Isolated from ICC169 infected mouse ‘a’ faeces 15 days post infection	PFGE profile same as for ICC168	This study
ICC169-407b15	Isolated from ICC169-407 infected mouse ‘b’ faeces 15 days post infection	PFGE profile same as for ICC168	This study
ICC169-496c3	Isolated from ICC169-496 infected mouse ‘c’ faeces 3 days post infection	PFGE profile same as for ICC168	This study
ICC169-496c6	Isolated from ICC169-496 infected mouse ‘c’ faeces 6 days post infection	PFGE profile same as for ICC168	This study
ICC169-496c10	Isolated from ICC169-496 infected mouse ‘c’ faeces 10 days post infection	PFGE profile same as for ICC168	This study
ICC169-496c13	Isolated from ICC169-496 infected mouse ‘c’ faeces 13 days post infection	PFGE profile same as for ICC168	This study
ICC169-496c15	Isolated from ICC169-496 infected mouse ‘c’ faeces 15 days post infection	PFGE profile same as for ICC168	This study
ICC180	ICC168 derivative *luxCDABE*Km2, Km^R^	pCROD1^−^	[41]
ICC180-P10	Isolated from ICC180 infected mouse faeces after passage through 10 mice	Differences in PFGE profile compared to ICC180, pCROD1^−^	This study
***Escherichia coli***			
MG1655	K-12 wild type, non-pathogenic		[22]
ER2507	K-12 derivative, F^−^, *ara*-14, *leuB6*, *fhuA2*, Δ(*argF*-*lac*)U169, *lacY1*, *glnV44*, *galK2*, *rpsL20*(Sm^R^), *xyl*-5, *mtl*-5, Δ (*malB*), *zjc*::Tn*5*(Km^R^), Δ (*mcrC-mrr*)_HB101_		New England BioLabs
ER2507 NPL	ΦNP lysogen		This study

**Figure 3:**
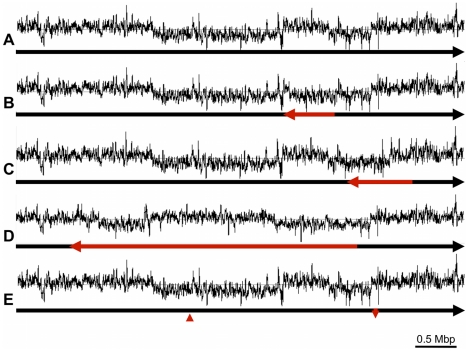
Chromosomal rearrangements identified in *C. rodentium* isolates ICC169-407 and ICC169-496. Deviations from (A) the sequence of the wild-type strain ICC168, are depicted. Rearrangements are highlighted in red and show: (B) genomic inversion between two copies of IS*Cro4*; (C) genomic inversion between two identical sequences encoding Elongation factor Tu; (D) genomic inversion between identical repeat sequences encoding the T3SS effector NleD and an adjacent transposase; (E) translocation of sequence from one location to another due to recombination between identical sequences in two Rhs elements. The GC deviation (G−C)/(G+C) plot for each genome orientation is shown above each sequence.

With the aim of pinpointing the rearrangement ‘break points’ we sequenced the genomes of two of the recombinant *C. rodentium* isolates, ICC169-407 and ICC169-496. By mapping 454 paired end sequences for these two isolates to the genome of the reference, wild-type strain ICC168, we identified positions where the 454 sequence pairs mapped to sequences either in the wrong orientation with respect to each other, or mapped to distant sites on the reference genome. These data highlighted four rearrangements within the sequenced genomes of ICC169-407 and ICC169-496 (Figures 3B–E). Three of these were large independent genomic inversions mediated by homologous recombination between two copies of different identical repeat sequences: i) IS*Cro4* (Figure 3B); ii) genes encoding Elongation factor Tu (Figure 3C); and iii) the T3SS effector NleD and the adjacent transposase (Figure 3D). It is of particular note that the IS*Cro4-*mediated 0.59 Mb inversion (Figure 3B) largely corrects the switch in GC deviation caused by the inversion identified in the ICC168 genome sequence (see above and Figure 3A). The fourth rearrangement in ICC169-407 and ICC169-496 can be explained by a double cross-over recombination event between two almost identical rearrangement hot spot (*rhs*) elements which could result in the translocation shown in Figure 3E. This provides biological evidence that *rhs* can diversify through intra-specific recombination, as previously speculated [27].

To confirm the nature of the rearrangements in ICC169-407 and ICC169-496 we used PCR to amplify sequences spanning the recombination points. Using different combinations of the primers NKP135-NKP150 (Table S2), we confirmed that all of the identified genome configurations (shown in Figures 3A–E) were present in genomic DNA preparations from a single culture of each isolate. These data indicate that the genome rearrangements observed are reversible and actively ‘flipping’ between one genome orientation and another, which suggests that the genome of *C. rodentium* is in a constant state of flux.

### Evidence for functional gene loss through disruption by mobile genetic elements and pseudogenisation

Our analyses showed that collectively, IS elements and prophages were responsible for 22% of the pseudogenes in the *C. rodentium* ICC168 genome, with the insertion of five out of the ten prophages disrupting accessory and core genes [20]. Additional analysis of the mobile genetic element insertions, indicated that several of them may have resulted in phenotypic alterations crucial to the evolution of *C. rodentium* which led us to investigate further. Of particular note are the prophages CRP28 and CRPr20, the insertions of which have disrupted gene clusters for the biosynthesis of two distinct flagellar systems in *C. rodentium*. CRPr20 has inserted into one of the gene clusters that encode the conventional Flag-1 flagellar system found in most members of the enterobacteriaceae whereas CRP28 has inserted into the Flag-2 ancestral flagellar cluster, which is found in some *E. coli* strains but is absent from *Salmonella* and most other enteric bacteria [28], [29].

The insertion by CRP28 has resulted in the deletion of the Flag-2 *lfiH* gene, encoding a putative flagellar assembly protein, and truncation of the two flanking genes, *lfiG* and *lfiI* encoding predicted flagellar switch (C-ring), and flagellar export and assembly proteins respectively [29]. The remainder of the Flag-2 cluster genes remain largely intact, although *lfgF* (encoding a flagellar rod protein) and another gene within this cluster carry point mutations generating premature stop codons.

CRPr20 has inserted into the 3′ end of *fliC*, deleting the last six codons of the flagellin gene of Flag-1. Our analysis indicated that the truncated *fliC* may encode a protein with an altered C-terminus which could mean that flagellin was still synthesised and secreted, but not polymerised. However, as in Flag-2, in addition to the prophage insertion there are other significant disruptions in the Flag-1 flagellar biogenesis genes in the form of a deletion event which has removed *flgD,E,F,G,H* and *I*, and truncated *flgC* and *flgJ*, genes required for rod and hook formation and assembly [28]. There is also an IS element insertion in the gene encoding the flagellar assembly regulator and chaperone, FlgN [30]. The remaining Flag-1 genes appear intact.

In transmission electron microscopy (TEM) studies of *C. rodentium* ICC169 we saw no evidence of basal bodies, and there was no evidence of flagella in culture supernatants or lysates (data not shown). This is consistent with previous reports showing that there was no detectable flagellin in *C. rodentium* DBS100 and that the organism is non-motile [31]. Flagella are targeted by the TLR5 receptor of the innate immune system and the lack of flagella in *C. rodentium* could facilitate escape or modulation of any inflammatory response following infection. Most other bacteria belonging to the family Enterobacteriacae express functional flagella. Notable exceptions are *Shigella*, *Salmonella* Gallinarum and *Salmonella* Pullorum [32], which are all host adapted. Flagella may play a key role in environmental survival and the ability to survive without motility can be considered further evidence of host restriction.

Prophages CRP38, CRP99 and CRP49 were also found to have inserted within genes. CRP38 has inserted into a gene of unknown function, whilst the insertion of CRP99 has disrupted ROD_08971 that is predicted to encode a putative large repetitive protein showing significant homology to a Type I secreted large repetitive protein in *S.* Typhi, and a putative haemagglutinin/haemolysin-related protein in *Ralstonia solanacearum* [20]. CRP49 has inserted into *gatD* (encoding galactitol-1-phosphate dehydrogenase), which is essential for the metabolism of galactitol by *E. coli* [33], and found within the otherwise intact galactitol utilisation operon. Consistent with this, we found that *C. rodentium* is unable to grow in minimal medium with 0.5% galactitol as its sole carbon source (data not shown).

Since the same patterns of prophage-mediated insertional inactivation are seen in EX-33 it is clear that prophages either contribute to, or are driving the degenerative genome evolution of *C. rodentium.*

### Plasmid pCROD1 is lost at high frequency

To investigate the stability of the plasmids, we determined the plasmid profiles of a range of *C. rodentium* ICC168 derivatives. *C. rodentium* strain ICC168 carries four plasmids, pCROD1-3 and pCRP3 [20]. However, our profiling showed that whilst plasmids pCROD2 (39 kb), pCROD3 (4 kb) and pCRP3 (3 kb) were present in all isolates, the largest plasmid, pCROD1 (54 kb), was present in only five out of the nine isolates tested (Table 3). The plasmid profiling gels also showed that the relative intensities of the bands corresponding to each plasmid were equivalent across the isolates, with the exception of the 54 kb pCROD1 band for ICC168-474 where the intensity was greatly reduced (Figure S1), suggesting that pCROD1 is either present in a lower copy number in ICC169-474 or, considered more likely, has been lost from a proportion of the cells in that population. This was confirmed by PCR data, which showed that the large plasmid is only present in 50% of the population of ICC169-474 (two out of four gDNA extractions from cultures originating from individual colonies of the same generation), indicating that pCROD1 is lost at high frequency. This is perhaps surprising given that all four plasmids are retained by EX-33.

Plasmid pCROD1 is predicted to encode several potential virulence factors, including three putative autotransporters and a fimbrial operon [20], therefore its high frequency loss is a further indicator of ongoing genome evolution and adaptation to a new environment.

### Transcriptomic data reveals evidence for recent niche adaptation and prophage induction

To further investigate the impact of the *C. rodentium* mobile genetic elements, we performed a whole genome transcriptome analysis on *C. rodentium* strain ICC169-476, by RNA-seq using Illumina sequencing technology, to determine if the prophages or the pseudogenes found within the genome were expressed. This data revealed that 152 of the 182 pseudogenes in the ICC168 chromosome were transcriptionally active (Table S3; 73 have RPKM (Reads mapped Per Kilobase per Million reads) expression values of 1–9, 57 have values of 10 to 99, 12 have values >100). The expressed genes include *fliC* (RPKM value of 9), however, the fact that flagellin has not been detected for *C. rodentium* suggests that the transcript is not translated. The continued transcription of the majority of the *C. rodentium* pseudogenes, together with the low number of multiple mutations in them, was taken to suggest that the disruption of these genes were relatively recent events.

**Figure 4:**
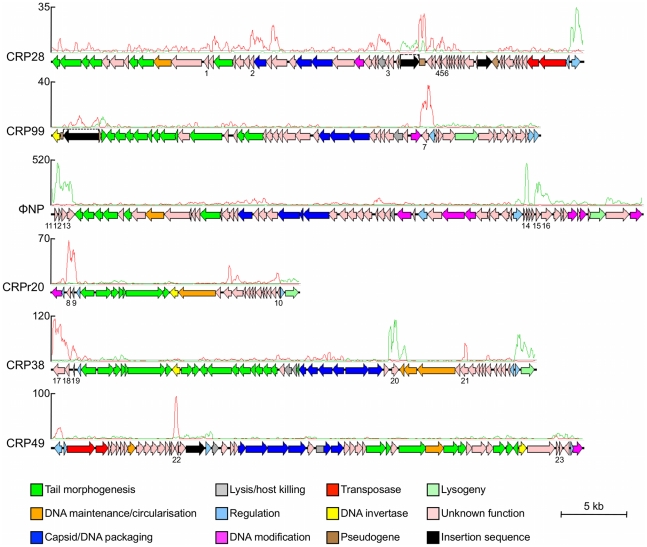
Genetic organisation of the *C. rodentium* prophages showing transcriptionally active genes. The genomes of each of the five intact prophages in the *C. rodentium* genome are shown aligned with mapped sequence reads for the whole genome transcriptome. The prophage remnant CRPr20 is also included due to its high similarity to CRP38 and the difficulty in mapping repetitive sequences. The RNA-seq data are represented as a plot showing the depth of sequences mapped to the forward strand (blue) and reverse strand (red) above each genome (window size = 200 bp). The majority of prophage genes, including those predicted to encode phage structural and lysis genes (see key), are expressed. Putative cargo genes can be identified by their relatively high levels of expression (numbered CDSs; see Table 4 for details). The scale bar indicates genome length. This figure was produced using Easyfig [67] and Artemis [68].

Surprisingly, our transcriptome data also revealed that the majority of the genes encoded on each of the five intact prophages (CRP28 55 out of 58 predicted CDSs, CRP99 52/55, ΦNP 65/65, CRP38 36/44, and CRP49 54/56) and the prophage remnant CRPr20 (21/29) were expressed under standard growth conditions (Figure 4, Table S3). This is unusual since for most prophages the structural and lysis genes are repressed in the lysogen [34], [35]. In addition to structural, lysis and regulatory genes, several of the other prophage-encoded genes showed relatively high levels of expression (Figure 4, Table S3). Bioinformatic analyses showed that in most cases these genes were in regions of aberrant GC content or they were encoded in regions that corresponded to known ‘cargo holds’ for non-essential genes by comparison with the genomes of well-characterised phages, such as Mu and P2. In most cases, the function of the genes in these transcriptionally highly active regions is unknown (Table 4), although putative damage-inducible and host-toxic membrane proteins encoded on ΦNP, CRPr20 and CRP38 were previously described, based on similarity to DinI and HokA respectively [20]. Some genes could be assigned putative products based on conserved protein domains such as transmembrane regions and signal peptides (Table 4). It is of note that the recently described effector NleK [36], encoded on CRP99 (ROD_09131), was not highly transcribed under the conditions tested. These data suggest that most of the *C. rodentium* prophages carry genes that represent known or novel lysogenic conversion functions.

**Table 4:** Highly expressed putative phage cargo genes.

Prophage	Number^a^	CDS ID^b^	Transcript RPKM Value	Product
CRP28	1	ROD_02501	43	hypothetical protein
	2	ROD_02551	51	hypothetical protein
	3	ROD_02681	37	putative membrane protein
	4	ROD_02733	53	putative membrane protein
	5	ROD_02751	39	putative exported protein
	6	ROD_02761	37	putative exported protein
CRP99	7	ROD_09341	72	putative lipoprotein
CRPr20^c^	8	ROD_19801	840	putative membrane protein
	9	ROD_19811	642	putative host toxic membrane protein
	10	ROD_20041	257	putative membrane protein
ΦNP	11	ROD_25751	605	putative damage-inducible protein
	12	ROD_25752	512	hypothetical protein
	13	ROD_25761	400	hypothetical protein
	14	ROD_26221	1415	hypothetical protein
	15	ROD_26231	596	hypothetical protein
	16	ROD_26241	213	hypothetical protein
CRP38	17	ROD_36461	299	putative exported protein
	18	ROD_36471	129	putative membrane protein
	19	ROD_36481	226	putative host toxic membrane protein
	20	ROD_36801	262	putative membrane protein
	21	ROD_36851	121	hypothetical protein
CRP49	22	ROD_47072	422	hypothetical protein
	23	ROD_47431	46	hypothetical protein

a Putative cargo gene number used in Figure 4.

b Prophage-encoded genes that have RPKM values >2x the average for that phage (average RPKM values for each prophage are: CRP28 = 18, CRP99  = 11, CRPr20 = 78, ΦNP  = 101, CRP38  = 38, CRP49  = 21). Genes predicted to be involved in the phage lytic or lysogenic cycle were excluded from this list.

c The prophage remnant CRPr20 is included in this list due to its high similarity to CRP38 and the difficulty in mapping repetitive sequences. The effector cargo genes encoded on CRPr13, CRPr17 and CRPr33 were described previously [20].

### *C. rodentium* prophages spontaneously excise and transpose within the genome

To determine if any of the *C. rodentium* prophages were capable of spontaneous excision, primers were designed to sequences at the ends of the integrated prophage genomes, facing outwards towards the prophage attachment sites (*attL* and *attR*). Using these primers (Table S2), DNA would only be amplified by PCR if the prophage excised from the host genome and circularised, bringing the primer pairs into the correct orientation with respect to each other.

PCR products were obtained for all the intact prophages CRP28, CRP99, ΦNP, CRP38, and CRP49, but not for the partial prophage CRPr20 (data not shown). Only a single sized PCR product was obtained for each of ΦNP and CRP38, which, on sequencing, showed that these phages precisely excised from the host genome. However, multiple PCR products of different sizes were amplified for CRP28, CRP99 and CRP49. These three prophages are all Mu-like [20] and, characteristically, carry phage transposition proteins, which can facilitate random transposition in the same way as for Mu and other transposable elements [37], [38]. The amplified PCR products obtained for each of CRP28, CRP99 and CRP49 were sequenced, revealing the terminal prophage sequences as well as a range of different intervening host genomic sequences. This is evidence of illegitimate excision, indicating that these phages are capable of random transposition and, if packaged, could be capable of specialised transduction.

We cloned and sequenced 235 of the intervening host genomic inserts for CRP99. The sequences for 133 of the inserts mapped to different chromosomal locations in the ICC168 genome sequence, 70 sequences mapped to regions in plasmid pCROD3 and 32 sequences mapped to plasmid pCRP3 (Figure S2), confirming that CRP99 was randomly transposing around the bacterial genome and taking adjacent bacterial DNA with it on excision from each genomic location. pCROD1 had been lost from the strain used as a template for this PCR, strain ICC169-476 (Table 3), however no sequences mapped to the 39 kb plasmid pCROD2, which was present in this strain (see above). The reason for this is not clear considering the depth at which we sampled independent insertions.

The size of the host chromosomal DNA inserts incorporated into the excised and circularised CRP99 genome varied from 16 bp to 3334 bp. This is comparable with the genome of phage Mu, which is found flanked by variable sequences of up to 150 bp of host DNA at the left hand end and up to 3 kb at the right hand end when packaged [38]. Significantly, of the plasmid derived sequences incorporated into CRP99, we were able to show that for 22 inserts in pCROD3 and 24 inserts in pCRP3 the whole plasmid had been incorporated into the circularised phage genome (3910 bp and 3172 bp respectively).

To our knowledge, this is the first description of entire plasmids being incorporated into a phage genome and provides intriguing evidence for the possibility of plasmid dissemination between bacteria via specialised transduction. Since neither of these plasmids have recognisable mobility markers of their own this may explain how they entered *C. rodentium*. It may also explain why plasmids similar in size to pCROD3 and pCRP3 are so successful and found in a wide range of different bacteria.

Analysis of the paired sequencing reads of EX-33 confirmed that ΦNP is spontaneously excising and circularising in both *C. rodentium* strains and showed evidence that the three Mu-like phages are also randomly transposing in the EX-33 genome.

### ΦNP produces virions capable of infecting and lysogenising *E. coli*

Considering the transcriptional activity of the *C. rodentium* prophage structural genes, TEM was used to examine culture supernatant to identify if functional virions were formed. Even in uninduced overnight cultures of strain *C. rodentium* ICC169-476, virions with an icosahedral head 70 nm in diameter, a 10 nm long neck and a contractile tail 115 nm long were visible. Although the majority of the virion tails observed were contracted, some with extended tails showed evidence of a base plate and tail fibres. This was the only virion morphology we observed, even with concentrated supernatant.

To determine if this phage had an extended host range, a variety of different bacteria available in our laboratories, including *Pseudomonas* sp., *C. freundii, Serratia* sp., *Pectobacterium* sp., *Yersinia enterocolitica, Salmonella* sv. and a range of pathogenic and non-pathogenic *E. coli* strains, were tested for susceptibility to infection using *C. rodentium* overnight culture supernatant. Plaques were observed on strains of *E. coli* K-12 and its derivatives, but no signs of infection were seen for any other bacterium tested.

The supernatant of an uninduced overnight culture of *C. rodentium* ICC169-476 produced between 10^5^ and 10^7^ pfu/ml when titrated on *E. coli* K-12 strains including MG1655. The plaques were turbid suggesting that the phage(s) present in the *C. rodentium* supernatant were able to lysogenise *E. coli* K12. This was confirmed using methods previously described [39]. Phage isolated from these plaques were propagated on *E. coli* K12 strain ER2507 to make high titre phage lysates and we cloned and sequenced DNA extracted from the phage virions in these lysates. The sequences obtained mapped to prophage ΦNP in the *C. rodentium* genome. Furthermore, random primed PCR performed on genomic DNA from *E. coli* ΦNP lysogen ER2507 NPL showed that ΦNP had inserted into an identical genomic location in *E. coli* ER2507 to the insertion site identified in *C. rodentium*, the *ssrA* tmRNA gene (data not shown, [20]).

**Figure 5:**
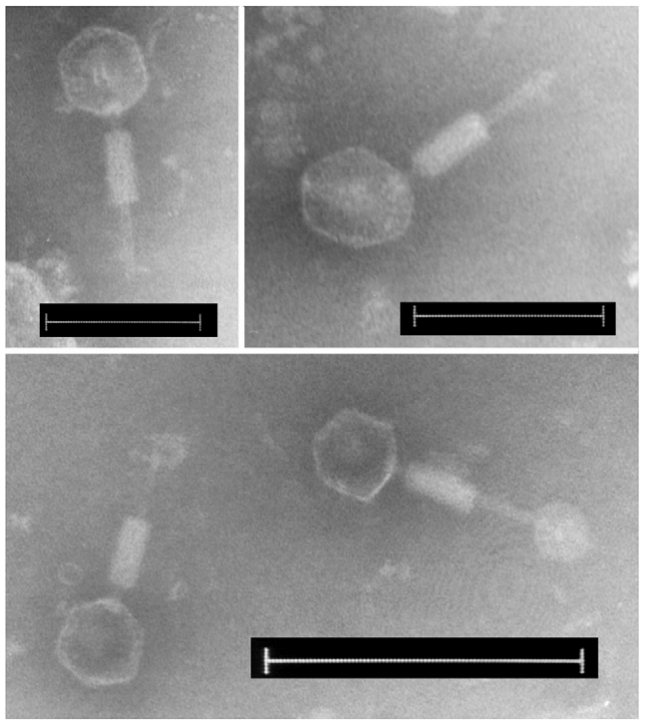
Electron micrographs of ΦNP negatively stained with phosphotungstic acid. All virions can be seen with contracted tails. Bars, 100 nm (top panels), 200 nm (bottom panel).

Host range studies using purified ΦNP lysates (propagated on *E. coli*) produced an identical infection pattern to that seen for the *C. rodentium* supernatant, and electron microscopy showed that ΦNP virions were identical in size and morphology to the virions observed in the supernatant. This morphology (shown in Figure 5) allowed classification of ΦNP into the order *Caudovirales* and family *Myoviridae* [40]. This may indicate that the only virions observed in the supernatant were those of ΦNP. Nevertheless, the possibility of the other *C. rodentium* prophages forming functional virions cannot be ruled out, as there could be functional virions other than ΦNP spontaneously formed and present in the supernatant of *C. rodentium*, for which a susceptible host has yet to be found.

### Genome flux in *C. rodentium* is a natural phenomenon

To determine if the genomic rearrangements observed *in vitro* were a natural phenomenon and had an impact on the ability of *C. rodentium* to infect its murine host, four isolates were tested to determine their virulence phenotypes. The isolates selected for murine infection were ICC169-407 and ICC169-496, which had both shown several band differences in PFGE profiles, along with two isolates which both had the same PFGE profile as ICC168: ICC169-476 which is missing the plasmid pCROD1; and the wild-type Nal^R^ strain ICC169.

**Figure 6:**
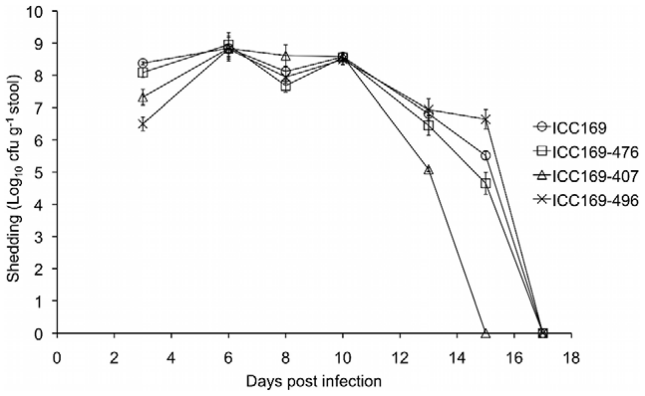
Mouse shedding of *C. rodentium*. Bacterial shedding in mouse faeces was monitored over the course of infection from individual mice. The mean count and standard deviation from groups of five mice infected with different *C. rodentium* isolates (see key) are shown.

Each of the different isolates of *C. rodentium* were able to colonise the gastrointestinal tract of the mouse, and all four groups of five mice showed a normal pattern of infection, as previously described [41]. Bacterial shedding in the faeces of individual mice was monitored over the course of the infection. We found no significant difference in the numbers of bacteria being excreted between the different groups of mice (Figure 6). The group infected by ICC169-407 had all cleared the infecting bacteria by day 15 post infection, however only mice infected with ICC169-496 had all mice in the group still shedding bacteria at this time point. Shedding of *C. rodentium* had ceased in all groups by day 17 post infection (Figure 6). On examination of the colons we found that there were no obvious differences in the extent of hyperplasia induced by the different isolates, with the average crypt lengths measuring from 211 µm (SD = 38 µm) to 240 µm (SD = 70 µm). These data indicate that neither the chromosomal rearrangements observed in ICC169-407 and ICC169-496, nor the natural loss of plasmid pCROD1, have any discernable effect on bacterial shedding or virulence in *C. rodentium*.

### Genome rearrangements occur *in vivo*

**Figure 7:**
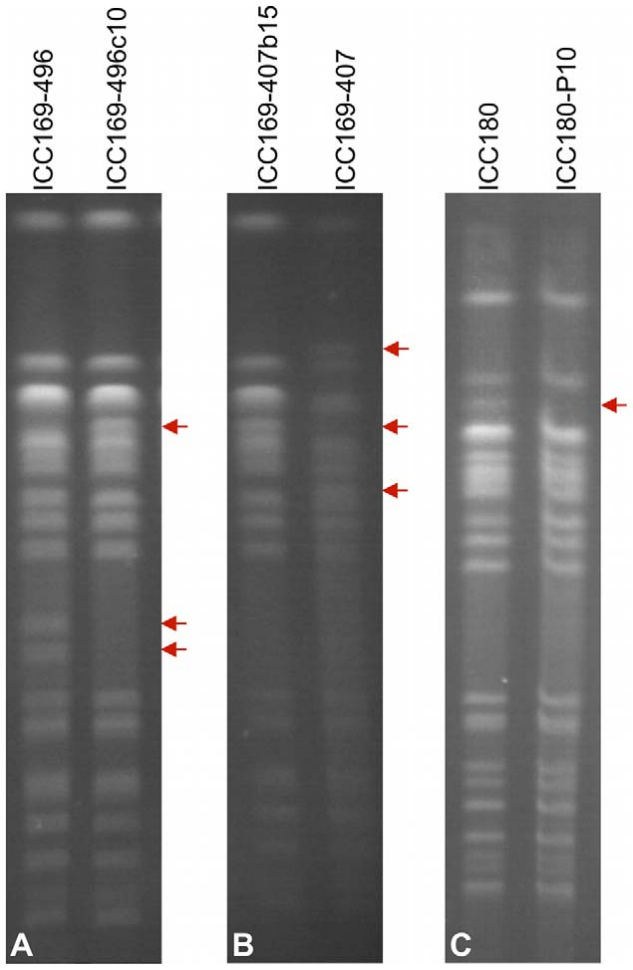
PFGE of *Xba*I-digested *C. rodentium* genomic DNA from isolates recovered before and after mouse inoculation. (A) PFGE profile of isolate ICC169-496 pre mouse inoculation (left) and isolate ICC169-496c10 recovered from mouse faeces 10 days post inoculation. (B) PFGE profile of isolate ICC169-407b15 recovered from mouse faeces 15 days post inoculation (left) compared to that of the infecting isolate ICC169-407 pre mouse inoculation (right). (C) PFGE profile of the pre mouse inoculation strain ICC180 compared to that for ICC180-P10 recovered from faeces after 10 passages though the mouse. Additional/missing bands are indicated by red arrows.

The effect of mouse passage on the genome architecture of *C. rodentium* was also determined. We performed PFGE analysis on selected ICC169 isolates obtained from mouse faeces at different times during infection and also from the colon and caecum, the organs colonised by this pathogen. These isolates are described in Table 3. PFGE showed that all the post-mouse passage isolates tested, displayed an identical banding pattern to each other and to the original strain ICC168 (as shown in Figure 2). This was even true for isolates taken from mice infected with the isolates ICC169-496 and ICC169-407, which display different PFGE patterns to ICC168 (Figures 7A and B, Figure 2). This may indicate that the different genome rearrangements in these isolates reverted to the original genomic configuration, that of the wild-type strain ICC168, on passage through the mouse. Alternatively, it is possible that a minor subpopulation with the wild-type genome conformation, that we have shown to exist within cultures of ICC169-496 and ICC169-407, was selected for within the mouse.

For all the strains tested in mice, the genomic rearrangements identified appear to be entirely neutral with regards to virulence and the progression of the infection. However, the fact that the alternative PFGE profiles seen for ICC169-407 and ICC169-496 revert to the original, ICC168-like, profile *in vivo*, indicates that this original genomic orientation may provide a fitness advantage in the murine host. The only observed phenotypic effect of genome rearrangement was for two ICC180 isolates (Table 3). ICC180-P10, isolated from mouse faeces after ten successive passages through mice by natural transmission from infected to naive animals through the faecal-oral route, had a different PFGE profile compared to the wild-type, pre-passage isolate ICC180 (Figure 7C). Interestingly, we found that the virulent phage ΦCR1, known to target lipopolysaccharide (LPS) as a receptor [42], was unable to infect the post-mouse passage isolate ICC180-P10, although it was able to form clear plaques on the wild-type ICC180 and all the other *C. rodentium* strains used in this study (data not shown). This data suggests that the genome rearrangement observed in ICC180-P10 may have affected LPS biosynthesis.

Our data indicate that genome instability is a feature of *C. rodentium in vivo*, as well as *in vitro* and that the genomic rearrangements observed are indicative of natural variation within a population. It is plausible that an invertible genome region may result in a differential expression of genes, which could allow rapid adaptation to different environments or stresses. This has been seen previously in *Campylobacter jejuni* where large-scale intra-chromosomal inversions, which were reversible, were associated with escape from infection by endogenous virulent phages on passage through the avian gut [43]. In addition, genomic rearrangements have previously been identified in strains of *Helicobacter pylori* [44], *Staphylococcus aureus* [45], *Pseudomonas aeruginosa* [46] and *E. coli* [47]–[50] during the course of human infection and appear to be linked to niche adaptation. Thus, in different environments, for example *in vivo* and *in vitro*, the dominant populations of *C. rodentium* could show different genomic arrangements, as demonstrated by the singular genomic conformation of post-mouse infection *C. rodentium* strains, despite the different genome arrangements of the infecting strains. However, further work is needed to understand the full impact of each genomic rearrangement on gene expression in *C. rodentium*, and to determine if this is a widespread phenomenon in other bacteria.

### Concluding remarks

We have shown that the genome of *C. rodentium* is in a state of considerable gene flux through large-scale, repeat-mediated recombination both *in vitro* and in the murine host, and also through the expansion of IS elements and the presence of several actively transposing prophages which are able to insert, apparently at random, throughout the chromosome and plasmids. Gene flux has also resulted in significant functional gene loss, particularly due to prophage and IS element insertions, which were fixed and invariant in all of the *C. rodentium* ICC168 derivatives we sequenced. The fact that almost identical patterns of gene loss can be seen in two lineages of *C. rodentium*, independently isolated from diseased mice on two different continents, a decade apart, is consistent with this occurring at the root of the evolution of this species and is likely to have played a significant role in the evolution of *C. rodentium* as an A/E pathogen of mice. One such example is the loss of flagella production through disruption of both flagella biogenesis systems, as flagella are known to be important elicitors of the innate immune system.

However, in addition to the loss of functions associated with virulence, *C. rodentium* has also lost metabolic capacity, for example we have shown that the operon encoding galactitol utilisation in ICC168 and EX-33 has been disrupted by prophage insertion, thereby limiting the available number of carbon sources that can be used by this bacterium. There are several examples now of other bacteria where loss of metabolic flexibility is associated with having recently changed niche [51]–[54].

It is clear from mouse infection studies that chromosomal rearrangements are a natural phenomenon and that, as might be expected, ongoing genome flux is largely neutral, not having had time for selection to play a role, and so having no discernable effect on bacterial shedding or virulence in the murine host. This included the loss of the large plasmid pCROD1, which, despite encoding two toxin-antitoxin addiction systems [20], our data shows is lost at high frequency.

We previously showed that many of the functions that confer *C. rodentium* with a common virulence strategy to EPEC and EHEC are located on horizontally acquired mobile genetic elements [20]. This, together with the large-scale genomic rearrangements and functional gene loss described in this study, suggests that *C. rodentium* has only recently emerged as a significant pathogen and is still adapting to its new lifestyle. Furthermore, the fact that *C. rodentium* is not known to cause disease in wild mice, only in laboratory rodents, may indicate that this pathogen emerged with the development of the mouse as a model organism and the large-scale captive breeding of small rodents. This would certainly explain the clonal nature of this species.

## Materials and Methods

### Ethics statement

This study was performed under project licence number 80/2099 approved by the UK Home Office and carried out in strict accordance with the UK Animals (Scientific Procedures) Act 1986. The Wellcome Trust Sanger Institute's Ethical Review Committee approved the research protocols used in this study.

### Bacterial strains and culture conditions

*C. rodentium* and *E. coli* strains were grown at 37°C in Luria–Bertani (LB) medium. For solid medium 1.5% agar was added, and for soft medium overlay (top agar) 0.15% agarose was used. When required, nalidixic acid (Nal) was added to LB to a final concentration of 50 µg/ml for selection. Phage buffer was composed of 10 mM Tris/HCl pH 7.4, 10 mM MgSO4, and 0.01% gelatin. The bacterial strains described in this study are listed in Table 3. The *C. rodentium* isolates ICC169-335, ICC169-407, ICC169-474, ICC169-476 and ICC169-496 all came from the same original stock of ICC169, but show different PFGE profiles and/or plasmid content.

### EX-33 genome sequencing and comparative analysis

The whole genome of *C. rodentium* strain EX-33 was sequenced on the 454/Roche GS FLX analyzer, with long-read GS FLX Titanium chemistry from a 3 kb insert paired end library prepared according to the manufacturer's specifications. A *de novo* assembly was produced from the generated sequence data using the 454/Roche Newbler assembly program (Software Release 2.1), which produced 27 scaffolds with an N50 scaffold size of 390,676 bp (largest scaffold size 699,126 bp) and 867 contigs with an N50 contig size of 9,811 bp (largest contig size 41,345 bp). The assembly consisted of 249,640 sequence reads (including 91,558 paired reads) totalling 43,628,532 bp, constituting a theoretical 8-fold coverage.

EX-33 was also sequenced on the Illumina GA II analyzer. A standard Illumina library was made with a 200 bp insert size and sequenced to a 54 bp read length using standard protocols [55], and a *de novo* assembly was produced using the Velvet assembly program. The optimal assembly was produced from a kmer length of 31. It generated 1,761 contigs with an N50 contig size of 4,177 bp (largest contig size 33,600 bp) from 8,751,150 sequence reads, constituting a theoretical 88-fold coverage.

The sequence data from the two sequencing platforms (individual 454 reads and consensus reads from the shredded Illumina assembly) were combined and assembled using the 454/Roche Newbler assembly program (Software Release: 2.3) into a consensus sequence of 382 total contigs (294 large contigs; N50 contig size, 38,722 bp) from 272,234 sequence reads totalling 54,256,007 bp, constituting a theoretical 10-fold coverage. Contigs were scaffolded using paired reads with an average pair distance of 2,998 bp into 40 scaffolds (N50 scaffold size, 244,370 bp) totalling 5,318,492 bp.

The EX-33 scaffolded contigs from the combined 454-Illumina assembly were ordered according to the ICC168 genome sequence (accession numbers FN543502 (chromosome), FN543503 (pCROD1), FN543504 (pCROD2), FN543505 (pCROD3) and AF311902 (pCRP3)) using ABACAS [56], and the annotation transferred from the reference genome.

Insertions/deletions in the EX-33 genome were identified by pairwise whole genome comparison of the ordered scaffolded contigs with the ICC168 genome sequence using BLASTN and visualised using the Artemis Comparison Tool [57]. Deletions from the EX-33 genome with respect to the ICC168 genome were confirmed by contiguated sequence spanning the syntenic regions in EX-33 and sequencing reads spanning each insertion/deletion region in the mapped coverage plot generated using SSAHA [58].

For SNP detection, the EX-33 454-Illumina combined assembly consensus sequence was shredded, resulting fragments were mapped by SSAHA and SNPs called with respect to the reference ICC168 genome and validated according to previously described protocols [59]. In addition, SNPs that were not located in repetitive sequences were validated manually, and only SNPs found in at least 5 sequencing reads, mapping to both strands, and present in at least 75% of the reads were passed as high-quality SNPs.

To identify gene flux and genomic rearrangements in the EX-33 genome, the paired Illumina sequencing reads were mapped to the ICC168 reference sequence using Maq (http://sourceforge.net/projects/maq/) and mismapping read pairs were identified using BamView [60].

### PFGE

DNA embedded in plugs was prepared using the CHEF Genomic DNA Plug Kit (Bio-Rad Laboratories, Hercules, CA, USA) from bacterial cells in suspension buffer (Bio-Rad Laboratories), grown to an optical density at an absorbance of 610 nm (OD_610_) of 1.3–1.4. Restriction digestion was performed with 30 U of *Xba*I (New England BioLabs) at 37°C overnight. Plugs were soaked in 0.5× TBE for 15 min at 4°C prior to electrophoresis. DNA fragments were resolved in 1% SeaKem Gold agarose (FMC Bioproducts, Rockland, ME, USA) in 0.5 x TBE buffer at 10°C, using a CHEF DR-III system (Bio-Rad Laboratories), running at a linear ramping factor of 2–68 s, pulse angle at 120°. The run length was 25 h at a constant voltage of 6 V/cm. DNA restriction patterns were assessed visually following ethidium bromide staining.

### Identification of recombination break points in *C. rodentium* isolates

The whole genomes of *C. rodentium* strains ICC169-407 and ICC169-496 were sequenced by paired-end 454 FLX pyrosequencing and assembled using the 454/Roche Newbler assembly program. For ICC169-407, contigs (1700 total contigs, 1355 large contigs; N50 contig size, 5,633 bp) were assembled from 290,987 sequence reads with an average read length of 168 bp, constituting a theoretical 9-fold coverage, contigs were scaffolded using paired reads with an average pair distance of 3,715 bp into 68 scaffolds (N50 scaffold size, 378,576 bp). For ICC169-496, contigs (4600 total contigs, 3081 large contigs; N50 contig size, 1,332 bp) were assembled from 243,094 sequence reads with an average read length of 162 bp, constituting a theoretical 7-fold coverage, contigs were scaffolded using paired reads with an average pair distance of 2,595 bp into 153 scaffolds (N50 scaffold size, 45,450 bp).

Scaffolded contigs were aligned with the ICC168 genome sequence using ABACAS. Read pairs with an insert size of at least 2 kb were mapped to the scaffold contigs using SSAHA and only read pairs that mapped uniquely and with maximum quality were selected. Recombination break points were found by BLASTN of the scaffold contigs against the genome of ICC168 and identifying single scaffold contigs that matched with two disparate regions of the reference genome, and also had reads spanning the putative point of recombination. The read pairs were then mapped to the reference genome, using SSAHA, and the break points were confirmed by a lack of reads spanning the corresponding region in the ICC168 genome sequence. Rearrangement break points were confirmed by PCR using primers designed to non-repetitive DNA sequences in the genome of ICC168 (Table S2).

### Carbon source growth curve

An overnight culture of *C. rodentium* ICC168 was seeded 1∶50 into 50 ml of minimal media consisting of M9 salts supplemented with either 1% glucose or 1% galactitol as a carbon source. The cultures were then incubated at 37°C with agitation at 200 rpm. The OD_600_ was measured every 60 min for 7 h using a Helios spectrometer (Thermo Scientific).

### Plasmid profile

*C. rodentium* plasmid content and sizes were assessed according to the method of Kado and Liu [61], and confirmed in different isolates by PCR of cultures originating from individual colonies of the same generation using the primers NKP111-NKP118 (Table S2).

### Transcriptomics

The whole genome transcriptome of ICC169-476 was sequenced using Illumina sequencing technology as previously described [62]. Removal of genomic DNA from the RNA sample and subsequent successful cDNA generation was confirmed by PCR using the four primer pairs NKP125-132 that generate amplicons internal to *C. rodentium* housekeeping genes (Table S2). Expression values were calculated as Reads mapped Per Kilobase per Million reads (RPKM) and recorded for each predicted CDS in the ICC168 genome (Table S3).

### Prophage excision/transposition detection

PCR analysis to detect spontaneous prophage excision and circularisation was performed using supernatant from an overnight culture of *C. rodentium* ICC169-476 or ICC169-407 as a template and primer pairs designed to sequences at the ends of the integrated prophage genomes, facing outwards towards the prophage attachment sites for each prophage (CRP28L and CRP28R, CRP99L and CRP99R, NPout1 and NPout4, CRP38L and CRP38R, CRP49L and CRP49R). The CRPr20 primers CRP20L and CRP20R were also used as a control. Primers are listed in Table S2.

To confirm prophage transposition, CRP99 PCR products were end repaired, gel purified and then cloned into *Sma*I cut pUC19 vectors. MegaX DH10B T1R electro-competent cells (Invitrogen) were used for the transformations, and transformants were selected on *Xgal*/IPTG (blue/white screen). Libraries were sequenced using standard forward and reverse primers. Sequences of at least 300 bp in length (of which approximately 200 bp mapped to one or other end of prophage CRP99) were mapped to the *C. rodentium* ICC168 genome sequence. Circular diagrams showing the insertion sites were made using DNAplotter [63].

### Phage characterisation

ΦNP virions were isolated from plaques formed on *E. coli* K-12 strain ER2507 after titration with chloroform-treated supernatant from an overnight culture of *C. rodentium* ICC169-476. Following plaque purification and further propagation on *E. coli* K-12 strain ER2507, DNA was extracted from high titre ΦNP lysates as previously described [42]. For cloning, ΦNP DNA and a pUC19 vector were digested with *Bam*HI prior to ligation. The ligated vector and insert were used to transform chemically competent *E. coli* DH5α cells, and the transformed cells selected, using a blue/white screen, purified and sequenced using standard primers.

The integration site of ΦNP in *E. coli* was determined by random primed PCR [64] on the *E. coli* ΦNP lysogen ER2507 NPL using the ΦNP specific primers NPL1 and NPR1, and the nested primers NPL2 and NPR2 respectively (Table S2). The resulting PCR products were sequenced and mapped to the *E. coli* K-12 MG1655 genome sequence (Accession number U00096) [22].

Transmission electron microscopy (TEM) and host range determination were performed as described previously [39].

### Murine infections

Female 6–8 weeks old C57BL/6 mice, purchased from Charles River (Margate, United Kingdom), were used to assess the virulence of different *C. rodentium* ICC169 isolates. All mice used in these studies came from colonies that were specific-pathogen free. Animals were housed in individually HEPA filtered cages with sterile bedding and free access to sterilised food and water. *C. rodentium* inocula were prepared by culturing bacteria overnight at 37°C in 100 ml of LB containing Nal. Cultures were harvested by centrifugation and resuspended in a 10% volume of PBS. Groups of five mice for each strain tested were orally inoculated using a gavage needle with 200 µl of the bacterial suspension. The viable count of the inocula was determined by retrospective plating on LB agar plates containing Nal. At selected time points post-infection, faeces were aseptically collected (100 mg faeces/ml PBS), serially diluted in PBS and plated on LB agar containing Nal. All plates were incubated overnight at 37°C. When all mice had stopped shedding bacteria the mice were sacrificed and colons removed. Small pieces of colonic tissue were fixed in 4% formaldehyde, then paraffin embedded, sectioned and stained with haematoxylin and eosin, for histological examination.

In addition, mice were orally inoculated with *C. rodentium* ICC180 as previously described and at the peak of infection (day 7 post-gavage) housed with naive mice to allow the natural transmission of ICC180 to occur via the faecal-oral route [65]. The natural transmission of ICC180 was followed by aseptic recovery of faecal samples from each animal at various time points after introduction. Mice infected in this way (termed passage 1, P1) were then housed with naive animals and the newly infected animals designated passage 2 (P2). This was continued until ICC180 had undergone ten successive passages from infected to naive mice, and ICC180-P10 was isolated from the faeces of passage 10 (P10) mice.

### Accession numbers

The EX-33 genome sequencing reads from both the Illumina and 454 platforms have been deposited in the Short Read Archive under the accession number ERS005106. The combined 454-Illumina assembly of the EX-33 contigs can be accessed from the website of the Wellcome Trust Sanger Institute (http://www.sanger.ac.uk/resources/downloads/bacteria/). The sequence and annotation of the fimbrial operon unique to EX-33 has been submitted to the EMBL/GenBank/DDBJ databases with the accession number FR715298.

The 454 sequencing reads for ICC169-407 and ICC169-496 have been deposited in the Short Read Archive under the accession numbers ERS004752 and ERS004750 respectively, and the ICC169-476 transcriptome Illumina sequencing reads can be found at ArrayExpress under accession number E-MTAB-502.

## Supporting Information

Figure S1 Plasmid profile of different *C. rodentium* isolates. Ethidium bromide-stained 0.7% agarose gel. *S. enterica* Typhimurium SL1344 (http://www.sanger.ac.uk/resources/downloads/bacteria/salmonella.html) was used as a control and marker; the sizes of the three plasmids in its genome are indicated. *C. rodentium* isolates ICC168, ICC169, ICC169-407 and ICC169-474 all have the same sized band at 54 kb, which corresponds to the large plasmid pCROD1. The intensity of this band is comparable for ICC168, ICC169 and ICC169-407, but for ICC169-474 the intensity is greatly reduced. ICC180 and ICC169-496 do not have this band. All the *C. rodentium* isolates show bands of a size corresponding to the other three plasmids, pCROD2 (39 kb), pCROD3 (3.9 kb) and pCR3 (3.2 kb). Chr  =  sheared chromosomal DNA bands. Plasmid sizes are indicated in kb.
(TIF)

Figure S2 Prophage CRP99 insertions in the genome of *C. rodentium*. 235 inserts from the circularised genome of prophage CRP99 genome were cloned, sequenced and mapped to the chromosome and plasmids of *C. rodentium* ICC168. 133 sequences mapped to the chromosome (left, green  =  complete insert sequence derived from paired end sequencing, blue  =  single read forward strand, red  =  single read reverse strand). 70 insertions were in plasmid pCROD3 and 32 were in plasmid pCRP3 (middle and right respectively). Paired end sequencing showed that the entirety of each plasmid was incorporated into the circularised CRP99 genome, and insertion sites were identified as direct repeats of 3–7 bp (shown in green on the two plasmids). For inserts with sequence data from one end only, insertion sites were inferred from the first 5 bp of sequence (shown in red for reads on the reverse strand and blue for reads on the forward strand). No insertions were detected in pCROD1 or pCROD2.
(TIF)

Table S1 Intragenic SNP differences between *C. rodentium* strains EX-33 and ICC168.
(DOC)

Table S2 Oligonucleotide primers used in this study.
(DOC)

Table S3 Transcription values for each predicted CDS in the genome of *C. rodentium* ICC168.
(XLS)
