# Supplementary figures and images for: Citrobacter rodentium is an Unstable Pathogen Showing Evidence of Significant Genomic Flux
Source: PLoS Pathog. 2011 Apr 7;7(4):e1002018. doi: 10.1371/journal.ppat.1002018 (PMC3072379; doi:10.1371/journal.ppat.1002018)

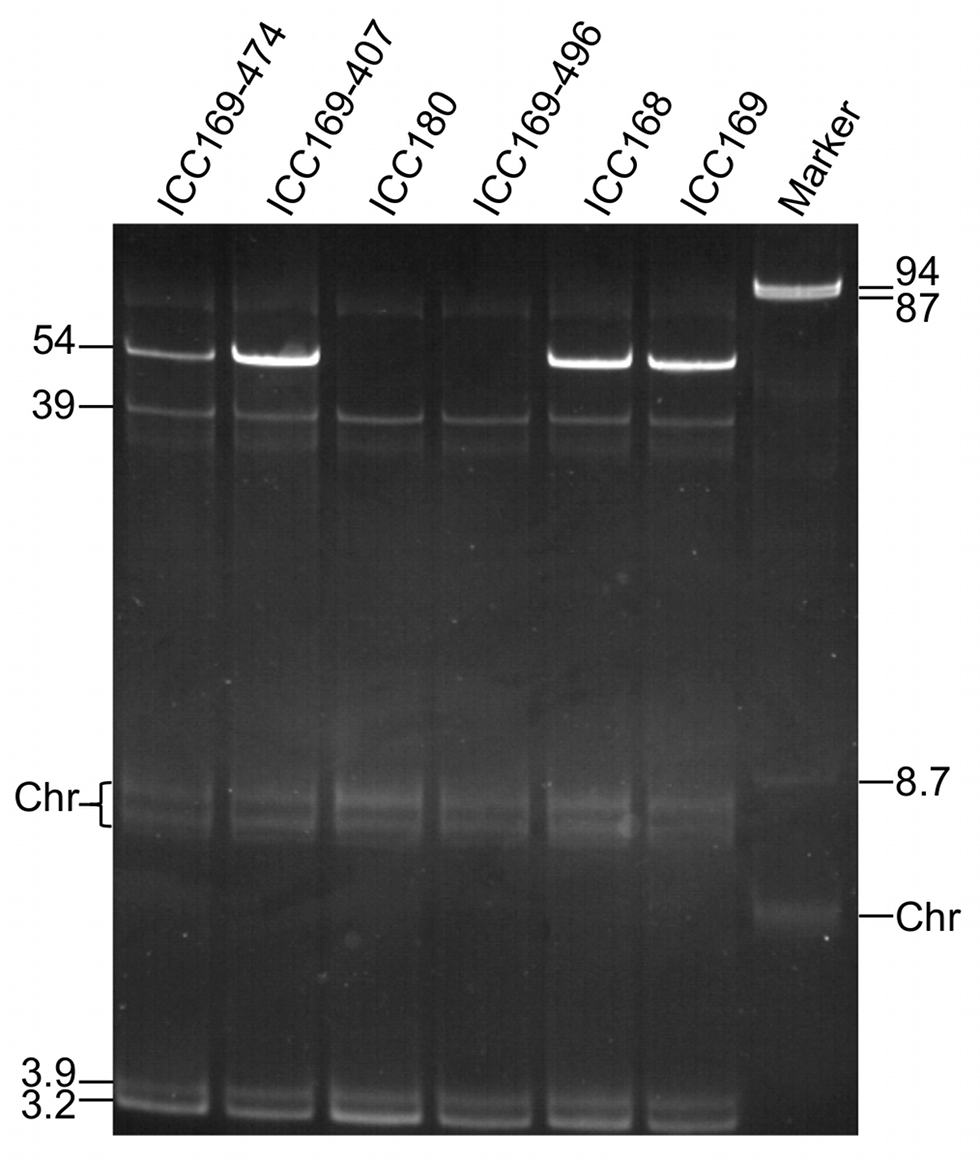

Supplement: Figure S1 — Plasmid profile of different C. rodentium isolates. Ethidium bromide-stained 0.7% agarose gel. S. enterica Typhimurium SL1344 (http://www.sanger.ac.uk/resources/downloads/bacteria/salmonella.html) was used as a control and marker; the sizes of the three plasmids in its genome are indicated. C. rodentium isolates ICC168, ICC169, ICC169-407 and ICC169-474 all have the same sized band at 54 kb, which corresponds to the large plasmid pCROD1. The intensity of this band is comparable for ICC168, ICC169 and ICC169-407, but for ICC169-474 the intensity is greatly reduced. ICC180 and ICC169-496 do not have this band. All the C. rodentium isolates show bands of a size corresponding to the other three plasmids, pCROD2 (39 kb), pCROD3 (3.9 kb) and pCR3 (3.2 kb). Chr = sheared chromosomal DNA bands. Plasmid sizes are indicated in kb. (TIF) [file ppat.1002018.s001.tif]

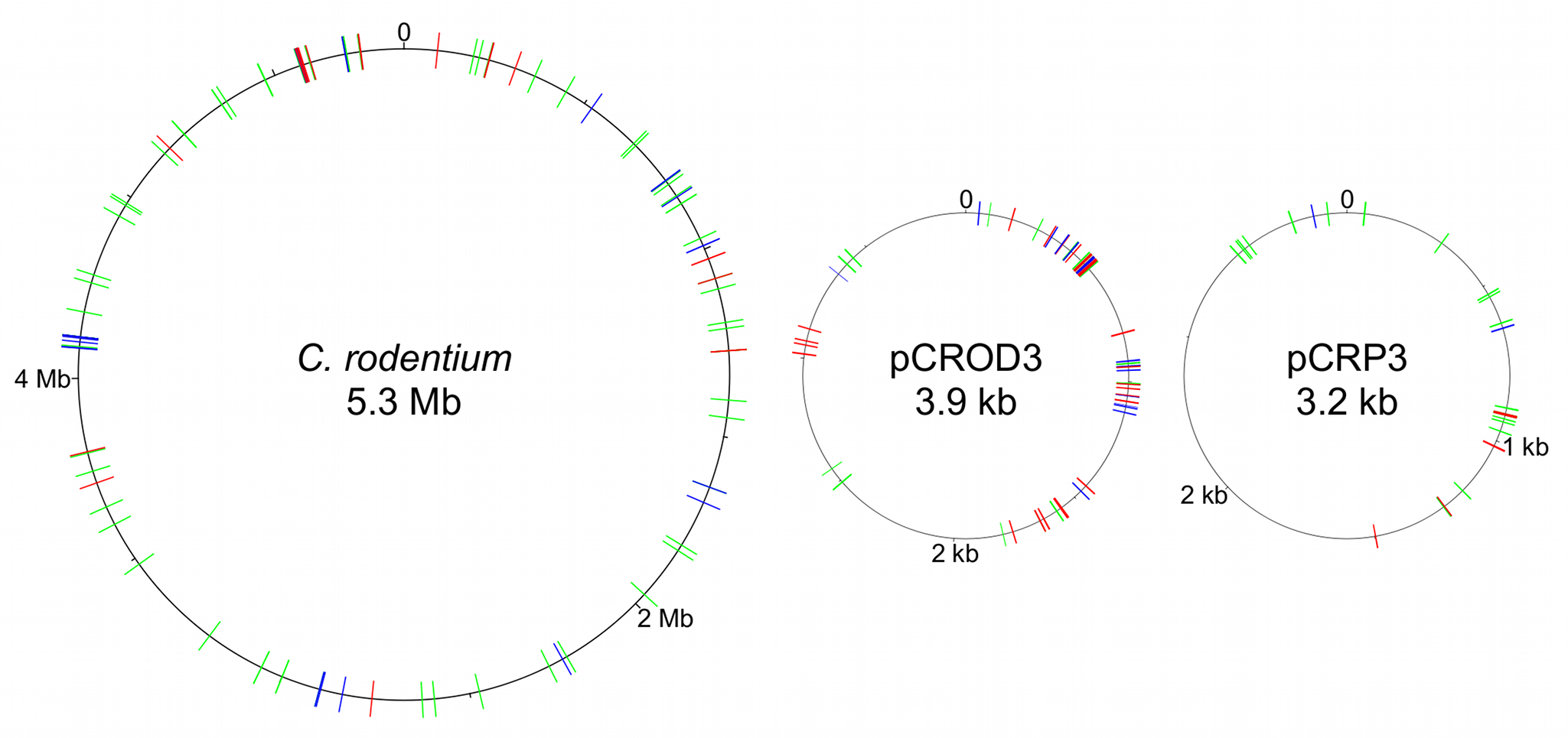

Supplement: Figure S2 — Prophage CRP99 insertions in the genome of C. rodentium. 235 inserts from the circularised genome of prophage CRP99 genome were cloned, sequenced and mapped to the chromosome and plasmids of C. rodentium ICC168. 133 sequences mapped to the chromosome (left, green = complete insert sequence derived from paired end sequencing, blue = single read forward strand, red = single read reverse strand). 70 insertions were in plasmid pCROD3 and 32 were in plasmid pCRP3 (middle and right respectively). Paired end sequencing showed that the entirety of each plasmid was incorporated into the circularised CRP99 genome, and insertion sites were identified as direct repeats of 3–7 bp (shown in green on the two plasmids). For inserts with sequence data from one end only, insertion sites were inferred from the first 5 bp of sequence (shown in red for reads on the reverse strand and blue for reads on the forward strand). No insertions were detected in pCROD1 or pCROD2. (TIF) [file ppat.1002018.s002.tif]
