# Supplementary material for: Citrobacter rodentium is an Unstable Pathogen Showing Evidence of Significant Genomic Flux
Source: PLoS Pathog. 2011 Apr 7;7(4):e1002018. doi: 10.1371/journal.ppat.1002018 (PMC3072379; doi:10.1371/journal.ppat.1002018)
Supplement: Table S1 — Intragenic SNP differences between C. rodentium strains EX-33 and ICC168. (DOC) [file ppat.1002018.s003.doc]

**Table S1. Intragenic SNP differences between *C. rodentium* strains EX-33 and ICC168.**

| **CDS ID** | **Gene** | **Number of SNPs** | | **Genomic context** | **Predicted product** |
| --- | --- | --- | --- | --- | --- |
|  |  | **Synonymous** | **Non-synonymous** |  |  |
| ROD_00531^v^ | *kefC* | 0 | 1 | core | glutathione-regulated potassium-efflux system protein |
| ROD_00971 ^v^ | *murC* | 1 | 0 | core | UDP-N-acetylmuramate:alanine ligase |
| ROD_02091 ^v^ | *metN* | 1 | 0 | core | D-methionine ABC transporter, ATP-binding protein |
| ROD_03501 ^v^ | *-* | 0 | 1 | core | putative MSF transporter protein |
| ROD_03891 | *-* | 3 | 1 | core* | putative adhesin autotransporter |
| ROD_04451 | *brnQ* | 1 | 0 | core* | branched chain amino acid transport system II carrier protein |
| ROD_05281 | *dnaX* | 0 | 1 | core* | DNA polymerase III subunits gamma and tau |
| ROD_05361 ^v^ | *-* | 0 | 2 | core | putative transport protein |
| ROD_05851 ^v^ | *-* | 0 | 1 | core | putative lipoprotein |
| ROD_05951 | *Fes* | 0 | 1 | core* | enterochelin esterase (ferric enterobactin esterase) |
| ROD_05961 | *entF* | 0 | 1 | core* | enterobactin synthetase component F |
| ROD_06031 ^v^ | *entE* | 0 | 1 | core | enterobactin synthetase component E |
| ROD_06941 ^v^ | *-* | 0 | 1 | core | putative esterase/lipase |
| ROD_08021 | *Adh* | 4 | 0 | core* | alcohol dehydrogenase |
| ROD_08801 ^v^ | *ItaE* | 0 | 1 | core | low specificity L-threonine aldolase |
| ROD_08971^p^ | *-* | 3 | 1 | GI2* | large repetitive protein (pseudogene) |
| ROD_09101 | *-* | 0 | 1 | CRP99* | putative phage tail protein |
| ROD_10801^p^ | *-* | 0 | 1 | IS*Cro3** | IS*Cro3* transposase (fragment) |
| ROD_11151 ^v^ | *-* | 0 | 1 | core | putative exported protein |
| ROD_11161 ^v^ | *-* | 1 | 0 | core | cytochrome b561 homolog |
| ROD_12741 ^v^ | *msrB* | 0 | 1 | core | peptide methionine sulfoxide reductase |
| ROD_14131 | *-* | 0 | 1 | core | major facilitator superfamily protein |
| ROD_15911 ^v^ | *-* | 1 | 0 | core | ABC transporter, permease protein |
| ROD_17381 | *trpH* | 0 | 1 | core | putative phosphoesterase |
| ROD_17681 | *adhE* | 9 | 0 | core* | aldehyde-alcohol dehydrogenase |
| ROD_17881 ^v^ | *nasB* | 0 | 1 | core | nitrite reductase [NAD(P)H] large subunit |
| ROD_20071^p, v^ | *fliC* | 0 | 1 | core | flagellin (pseudogene) |
| ROD_19911 | *A* | 8 | 2 | CRPr20* | putative phage DNA replication protein |
| ROD_19921 | *-* | 0 | 1 | CRPr20* | hypothetical prophage protein |
| ROD_20021 | *-* | 1 | 0 | CRPr20* | hypothetical prophage protein |
| ROD_20031 | *-* | 1 | 0 | CRPr20* | hypothetical prophage protein |
| ROD_21141 | *cbiF* | 0 | 1 | core | cobalt-precorrin-4 C(11)-methyltransferase (Cobalt- precorrin-3 methylase) |
| ROD_21291 ^v^ | *pduG* | 1 | 0 | core | propanediol utilization diol dehydratase reactivation protein |
| ROD_22621^p^ | *dusC* | 0 | 1 | core | tRNA dihydrouridine synthase C (pseudogene) |
| ROD_23491 ^v^ | *rcsD* | 1 | 0 | core | two-component system response regulator |
| ROD_23701 ^v^ | *ligA* | 1 | 0 | core | DNA ligase |
| ROD_23971 | *eutC* | 0 | 1 | core* | Ethanolamine ammonia-lyase light chain |
| ROD_24221 ^v^ | *-* | 0 | 1 | core | putative esterase |
| ROD_25621 ^v^ | *corB* | 0 | 1 | core | putative magnesium transport protein |
| ROD_25701 | *-* | 7 | 3 | GI6* | large repetitive protein |
| ROD_26301 ^v^ | *-* | 0 | 1 | ΦNP | putative prophage exonuclease |
| ROD_27341 | *-* | 0 | 1 | core* | major facilitator superfamily protein |
| ROD_28041 ^v^ | *fryC* | 1 | 0 | core | fructose-like specific PTS system EIIC component 1 |
| ROD_28321 | *-* | 0 | 1 | IS*Cro1** | IS*Cro1* transposase C |
| ROD_28741 | *mutH* | 0 | 1 | core* | DNA mismatch repair protein |
| ROD_29701 | *espF* | 1 | 0 | LEE* | T3SS effector protein EspF |
| ROD_30391 ^v^ | *-* | 0 | 1 | core | probable hydrolase |
| ROD_31861^p^ | *-* | 0 | 1 | GI9 | putative transposase (pseudogene) |
| ROD_32501 ^v^ | *ulaD* | 1 | 0 | core | 3-keto-L-gulonate-6-phosphate decarboxylase (L-ascorbate utilization protein D) |
| ROD_34221 ^v^ | *cts2R* | 0 | 1 | core | T6SS protein Cts2R |
| ROD_34561 ^v^ | *-* | 0 | 1 | core | putative membrane protein |
| ROD_35831 ^v^ | *-* | 1 | 0 | core | putative lipoprotein |
| ROD_36451^p^ | *-* | 1 | 0 | core | conserved hypothetical protein (pseudogene) |
| ROD_36711 | *-* | 1 | 2 | CRP38* | putative phage holin |
| ROD_36771 | *P* | 2 | 1 | CRP38* | putative large terminase subunit |
| ROD_36821 | *-* | 6 | 0 | CRP38* | putative prophage stability/partitioning protein |
| ROD_36831 | *A* | 10 | 1 | CRP38* | putative phage DNA replication protein |
| ROD_36941 | *-* | 2 | 3 | CRP38* | hypothetical prophage protein |
| ROD_40421 ^v^ | *-* | 1 | 0 | core | GntR-family transcriptional regulator |
| ROD_41801 | *-* | 0 | 2 | IS*Cro3** | IS*Cro3* transposase |
| ROD_42211 ^v^ | *mtlD* | 1 | 0 | core | mannitol-1-phosphate 5-dehydrogenase |
| ROD_42661^$, v^ | *dppD* | 0 | 1 | core | dipeptide ABC transporter, ATP-binding protein |
| ROD_42931 ^v^ | *-* | 0 | 1 | core | putative cytochrome C peroxidase |
| ROD_43401 ^v^ | *-* | 0 | 1 | core | conserved hypothetical protein |
| ROD_44021 ^v^ | *malT* | 0 | 1 | core | regulatory protein |
| ROD_44201 ^v^ | *envZ* | 1 | 0 | core | two-component sensor kinase |
| ROD_44801 | *tufA* | 1 | 0 | core* | Elongation factor tu (EF-Tu) |
| ROD_44841 | *-* | 1 | 0 | core* | putative chitinase |
| ROD_44941 ^v^ | *gspG* | 1 | 0 | core | putative T2SS protein G |
| ROD_47371 | *S* | 3 | 1 | CRP49* | phage tail fibre protein |
| ROD_47681 ^v^ | *-* | 0 | 1 | core | conserved hypothetical protein |
| ROD_47731 | *-* | 0 | 1 | IS*Cro1** | IS*Cro1* transposase C |
| ROD_47771 | *lifA3* | 1 | 1 | GI12* | LifA-like protein |
| ROD_50511 | *tktA* | 1 | 0 | core* | transketolase 1 |
| ROD_50591 ^v^ | *talA* | 1 | 0 | core | transaldolase A |
| ROD_p1_251 | *-* | 1 | 0 | pCROD1* | putative serine protease autotransporter |
| ROD_p4_51^$^ | *-* | 0 | 1 | pCRP3 | hypothetical protein |

^P^pseudogene in both ICC168 and EX-33

^$^pseudogene in EX-33, intact in ICC168

*SNP is in repetitive sequence or close to contig end in draft genome of EX-33

^v^ high quality, manually validated SNP
