# Supplementary material for: Citrobacter rodentium is an Unstable Pathogen Showing Evidence of Significant Genomic Flux
Source: PLoS Pathog. 2011 Apr 7;7(4):e1002018. doi: 10.1371/journal.ppat.1002018 (PMC3072379; doi:10.1371/journal.ppat.1002018)
Supplement: Table S2 — Oligonucleotide primers used in this study. (DOC) [file ppat.1002018.s004.doc]

**Table S2. Oligonucleotide primers used in this study.**

| **Name** | **Sequence (5’ – 3’)** | **Comments** |
| --- | --- | --- |
| NKP135 | GAGCTTCGGTATTCGCACTC | F primer 1 to detect *tuf* inversion, internal to ROD_37711 |
| NKP136 | AACACCTGGTTGGGATGAAG | R primer 1 to detect *tuf* inversion, internal to ROD_37751 |
| NKP137 | CGGTCAGTACGGTCATGTTG | F primer 2 to detect *tuf* inversion, internal to ROD_44791 |
| NKP138 | ACGGTGGAAGATGACCACTC | R primer 2 to detect *tuf* inversion, internal to ROD_44811 |
| NKP139 | ACCGTTCATGCAGTCCTACC | F primer 1 to detect *rhs* recombination, internal to ROD_40651 |
| NKP140 | AAGCCTCACCATCCAATCTG | R primer 1 to detect *rhs* recombination, internal to ROD_40751 |
| NKP141 | TCAAAAATGCGAGTCAGACG | F primer 2 to detect *rhs* recombination, adjacent to ROD_19231 |
| NKP142 | AACCCACTAACCCCCATAGC | R primer 2 to detect *rhs* recombination, internal to ROD_19281 |
| NKP143 | ATGGTGCGTTAAGGTCTTGC | F primer 1 to detect IS*Cro4* inversion, internal to ROD_30271 |
| NKP144 | CCGAGAAACTCAAGCAGGAC | R primer 1 to detect IS*Cro4* inversion, internal to ROD_30301 |
| NKP145 | CATCGACATAACGACCATCG | F primer 2 to detect IS*Cro4* inversion, internal to ROD_35961 |
| NKP146 | TCGCGGATATTCATCTTTCC | R primer 2 to detect IS*Cro4* inversion, internal to ROD_35981 |
| NKP147 | GCCGGAGTTTAAGTCCTTCC | F primer 1 to detect *nleD* inversion, internal to ROD_38641 |
| NKP148 | AACGTCATGCCTTCCAGTTC | R primer 1 to detect *nleD* inversion, internal to ROD_38711 |
| NKP149 | ATGCGATTCCCTGAATGAAG | F primer 2 to detect *nleD* inversion, internal to ROD_05741 |
| NKP150 | GATAGCGCCTCGTTTCAGAC | R primer 2 to detect *nleD* inversion, internal to ROD_05481 |
| NKP111 | CGCACAACTGAACCTTATGC | F primer for detection of plasmid pCROD1 |
| NKP112 | TTATAATCCACCGCCAGAGC | R primer for detection of plasmid pCROD1 |
| NKP113 | TACCCTTTTGCGACGATTTC | F primer for detection of plasmid pCROD2 |
| NKP114 | GGCTGGACTAAAAACGCTTG | R primer for detection of plasmid pCROD2 |
| NKP115 | ACACGGATGATCTCGCTTTC | F primer for detection of plasmid pCROD3 |
| NKP116 | TTTTTCCCTTTGTCCGACTG | R primer for detection of plasmid pCROD3 |
| NKP117 | ATCGCGTACCACTTTTCGAC | F primer for detection of plasmid pCRP3 |
| NKP118 | CCTGCTTCTGGCTAAACCAC | R primer for detection of plasmid pCRP3 |
| NKP125 | GGCGTTTGAATACGGTTACG | F primer internal to ROD_07231 (*sucA*) |
| NKP126 | GACGCAGCATGTGATACACC | R primer internal to ROD_07231 (*sucA*) |
| NKP127 | GTGGTGAACAATGACGATGC | F primer internal to ROD_00011 (*thrA*) |
| NKP128 | CAGGTTAAACGGCTCTTTCG | R primer internal to ROD_00011 (*thrA*) |
| NKP129 | ATGCTGTTTGAGACCGAAGG | F primer internal to ROD_40261 (*dnaN*) |
| NKP130 | CTTCTCGTTGGACAGGATCG | R primer internal to ROD_40261 (*dnaN*) |
| NKP131 | TCAGCGAAATTCTGGATAACG | F primer internal to ROD_21581 (*hisD*) |
| NKP132 | TCAGAACGGTGGAAAAGAGC | R primer internal to ROD_21581 (*hisD*) |
| CRP28L | CAGGTTGAGCGCAATGAC | LHS primer to detect circularised prophage CRP28 DNA |
| CRP28R | CAGCATGAGCGCCTACTG | RHS primer to detect circularised prophage CRP28 DNA |
| CRP99L | CGAACGGGAGCAGATTG | LHS primer to detect circularised prophage CRP99 DNA |
| CRP99R | CAGTGGATTTACAGCGTGG | RHS primer to detect circularised prophage CRP99 DNA |
| CRP20L | GCTTCTCAACACTGGAAGC | LHS primer to detect circularised prophage CRP20 DNA |
| CRP20R | GTTAAATCCGCTTGGTGGAG | RHS primer to detect circularised prophage CRP20 DNA |
| NPout1 | CCTTATCGATATTGGGTCGAGAA | RHS primer to detect circularised prophage ΦNP DNA |
| NPout4 | CAATCATTGCTATCGCATTCC | LHS primer to detect circularised prophage ΦNP DNA |
| CRP38L | GGCGTTCACTCTTCAGAAGG | LHS primer to detect circularised prophage CRP38 DNA |
| CRP38R | CGCAACCATTCAGCAAAC | RHS primer to detect circularised prophage CRP38 DNA |
| CRP49L | CTACTCATCCAGTCATCACCTG | LHS primer to detect circularised prophage CRP49 DNA |
| CRP49R | CAAATAAGTTGCGTATCTGCG | RHS primer to detect circularised prophage CRP49 DNA |
| NPL1 | GGGCTCAGAAGTGATGTTCTTC | RHS primer 1 for Random primed PCR out of ΦNP |
| NPL2 | GCTAGACGACTACCCACGGAC | RHS nested primer for Random primed PCR out of ΦNP |
| NPR1 | GTGTCGATCAGGTAAGAATTTTTG | LHS primer 1 for Random primed PCR out of ΦNP |
| NPR2 | TTGTCCCGCTACCGTCTG | LHS nested primer for Random primed PCR out of ΦNP |
